# SLM Magnesium Alloy Micro-Arc Oxidation Coating

**DOI:** 10.3390/ma17204988

**Published:** 2024-10-12

**Authors:** Xuejie Yue, Kangning Xu, Shuyi Wang, Hengyan Liu, Shiyue Guo, Rusheng Zhao, Gaopeng Xu, Hao Wang, Xuezheng Yue

**Affiliations:** 1 School of Civil Engineering and Architecture, Xinxiang College, Xinxiang 453000, China; 2 Department of Material Science and Chemical, University of Shanghai for Science and Technology, Shanghai 200093, Chinapres.boss@foxmail.com (S.W.);; 3 Hubei Longzhong Laboratory, Wuhan University of Technology, Xiangyang 441000, China; 4 Tokyo Metropolitan University, Tokyo 192-0397, Japan; 5 Institute of Metal Research, Chinese Academy of Sciences, Shenyang 110016, China; haowang7@usst.edu.cn

**Keywords:** selected laser melting, magnesium alloys, micro-arc oxidation, wetting angle, corrosion resistance

## Abstract

In this study, we utilized Selective Laser Melting (SLM) technology to fabricate a magnesium alloy, and subsequently subject it to micro-arc oxidation treatment. We analyzed and compared the microstructure, elemental distribution, wetting angle, and corrosion resistance of the SLM magnesium alloy both before and after the micro-arc oxidation process. The findings indicate that the SLM magnesium alloy exhibits surface porosity defects ranging from 2% to 3.2%, which significantly influence the morphology and functionality of the resulting film layer formed during the micro-arc oxidation process. These defects manifest as pores on the surface, leading to an uneven distribution of micropores with varying sizes across the layer. The surface roughness of the 3D-printed magnesium alloy exhibits a high roughness value of 180 nanometers. The phosphorus (P) content is lower within the film layer compared to the surface, suggesting that the Mg_3_(PO_4_)_2_ phase predominantly resides on the surface, whereas the interior is primarily composed of MgO. The micro-arc oxidation process enhances the hydrophilicity and corrosion resistance of the SLM magnesium alloy, thereby potentially endowing it with bioactivity. Additionally, the increased surface roughness post-treatment promotes cell proliferation. However, certain inherent defects present in the SLM magnesium alloy samples negatively impact the improvement of their corrosion resistance.

## 1. Introduction

Selective Laser Melting (SLM) is a proprietary powder bed fusion technology that employs a high-power laser and CAD data to selectively melt and fuse pre-dispersed powder particles in a layer-wise manner within designated areas [1,2]. The term “laser” in SLM denotes the use of a laser for the treatment process, “melting” describes the phase transition of the particles, and “selective” signifies that only a specific subset of the powder is subjected to processing. A comprehensive SLM system comprises a processing laser, a build platform, an automated powder distribution system, monitoring software, and essential auxiliary components [3]. Furthermore, the SLM process encompasses a variety of physical phenomena, including absorption, reflection, radiation, and heat transfer, as well as phase transformations, the dynamic interface between solid and liquid states, fluid flow induced by surface tension gradients, mass transfer within the melt pool, and chemical reactions [4]. SLM is an efficacious methodology that substantially diminishes production timeframes and mitigates the expenses inherent in the fabrication of high-value components. This technology confers the benefits of design flexibility, an extensive array of material options, minimized material wastage, and the obviation of custom mold requirements [5]. At present, SLM technology, owing to its distinctive advantages, has been extensively implemented across various sectors, including aerospace and medical implantology [6,7].

Biodegradable metals, such as magnesium alloys, have been extensively utilized as temporary implants in the fields of cardiovascular and orthopedic medicine [8]. Magnesium, which naturally occurs in bone tissue, can facilitate the formation of new bone [9]. Magnesium alloys exhibit mechanical properties, including density and elastic modulus, that closely resemble those of natural bone. This similarity can substantially mitigate the stress shielding effect and aid in the prevention of secondary fractures [10]. Moreover, once the objective of facilitating bone healing has been met, magnesium alloys can gradually dissolve and be absorbed by the body, thereby eliminating the need for a subsequent surgical procedure [11]. However, the high chemical reactivity of magnesium alloys makes them susceptible to rapid degradation within the body, which can result in a variety of complications, including a decrease in the mechanical strength of the implant and tissue inflammation due to hydrogen gas evolution [11,12].

Consequently, it is essential to employ suitable surface modification strategies to decelerate the corrosion rate of magnesium alloys. The array of surface modification techniques encompasses electrochemical deposition [13], micro-arc oxidation (MAO) [14], chemical conversion [15], and dip coating [16]. Micro-arc oxidation predominantly employs alkaline electrolyte systems, a choice often attributed to their low environmental impact and rapid film growth rates. These alkaline electrolyte systems are categorized into silicate, phosphate, and aluminate systems. The micro-arc oxidation (MAO) process has advanced from anodic oxidation techniques, offering numerous benefits including excellent corrosion resistance, robust adhesion, cost-effectiveness, and satisfactory wear resistance. Consequently, the MAO process has been extensively applied to magnesium alloys [14]. Hui Tang et al. [17] developed a hydroxyapatite coating on the surface of a AZ31 magnesium alloy via the micro-arc oxidation process, thereby enhancing its biodegradability. Mohsen Ghafarzadeh et al. [18] fabricated a dual-layer coating system consisting of micro-arc oxidation and polyglycerol sebacate (PGS) on a AZ91 magnesium alloy. This coating system not only enhanced the corrosion resistance but also significantly improved the biocompatibility of the biodegradable AZ91 alloy. Junxiu Chen et al. [19] incorporated copper (Cu) into the micro-arc oxidation process of magnesium alloys and discovered that the resulting Cu-MAO coating demonstrated superior corrosion resistance, antibacterial properties, and biocompatibility.

Current research on the micro-arc oxidation of magnesium alloys predominantly concentrates on those alloys produced through conventional casting methods. In contrast, magnesium alloys manufactured via Selective Laser Melting (SLM) are also candidates for surface treatment via micro-arc oxidation, albeit with a lesser extent of research dedicated to this process. Consequently, investigating the application of micro-arc oxidation technology to SLM-fabricated magnesium alloys and assessing the properties of the ensuing coatings is of considerable significance. In this study, the micro-arc oxidation electrolyte, composed of Na_3_PO_4_, KOH, and glycerol, is applied at a fixed voltage of 350 V for a duration of 30 min to evaluate the performance of the coating on magnesium alloys processed with SLM technology.

## 2. Experiment

### 2.1. Sample Preparation

**Figure 1:**
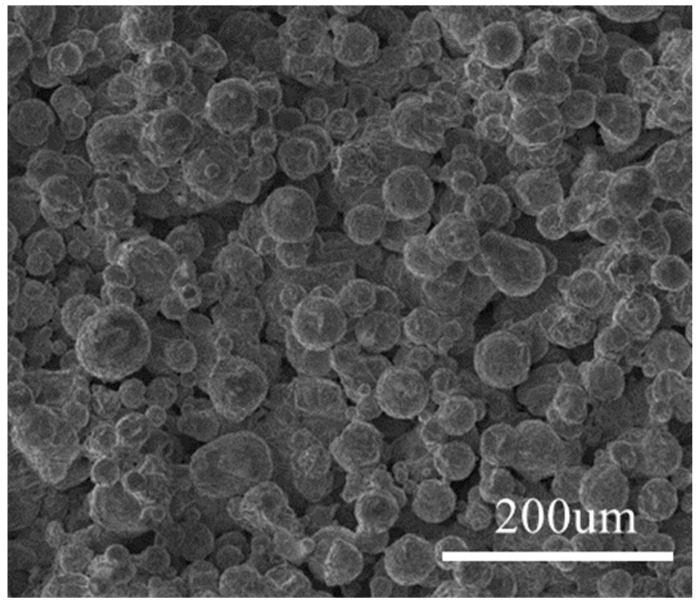
AZ91D magnesium alloy powder.

The gas-atomized AZ91D magnesium alloy powder was provided by Tangshan Weihao, Co., Ltd. Hebei, China. The particle size distribution spanned from 5 μm to 70 μm with volume-based D-values specifically noted as D10 = 8.1 μm, indicating 10% of the particles were smaller than this size; D50 = 22.4 μm signified the median particle size; and D90 = 47.5 μm signified that 90% of the particles were smaller than this dimension [20] as depicted in Figure 1, which shows the surface morphology images of the SLM magnesium alloy. The surface of the untreated sample was covered with numerous fine spherical particles, representing the unmelted metal powder particles. The average diameter of these powder particles was approximately 37 μm. The chemical composition of the powder comprised Al (~8.89 wt%), Zn (~0.45 wt%), Mn (~0.19 wt%), and Mg (balance), as specified by the manufacturer. The SLM (Selective Laser Melting) process utilized the EOSINT M280 machine from EOS GmbH, Krailling, Germany, with the following process parameters: a laser power of 170 W, scanning speed of 1250 mm·s^−1^, spot diameter of 100 μm, layer thickness of 30 μm, and scanning spacing of 60 μm. The entire forming process was conducted in an inert gas atmosphere, utilizing argon gas as a protective measure. To mitigate thermal stresses generated during the SLM process, which were due to rapid cooling rates, the formed samples underwent stress-relief annealing in a high-temperature vacuum furnace, followed by furnace cooling. The dimensions of the samples were 15 mm in length, 15 mm in width, and 4 mm in thickness.

During the micro-arc oxidation (MAO) process, the PSI 9000 2U series DC power supply produced by the EA Company in Helmholtzstr, Germany was used in manual mode and constant voltage mode in this experiment. The magnesium alloy samples served as the anode, with stainless steel plates acting as the cathode. The preparation conditions for the MAO coating were as follows: the applied voltage was 350 V, the oxidation duration was 30 min, and the electrolyte was composed of 10 g/L Na_3_PO_4_, 2 g/L KOH, and 10 mL/L glycerol based on our previously conducted research. Throughout the MAO process, the temperature of the solution was carefully controlled to remain within the range of 10–30 °C, achieved through vigorous stirring and the use of a water-cooling system.

### 2.2. Characterization and Performance Testing of the Micro-Arc Oxidation (MAO) Coating

The phase composition of the micro-arc oxidation coating was examined utilizing an advance-type X-ray diffractometer (XRD). The XRD analysis was conducted using a voltage of 40 kV and a current intensity of 40 mA, with a precise step size of 0.05° and a swift scan rate of 4°/min, comprehensively scanning in the range of 20° to 80°, ensuring comprehensive characterization of the sample. The surface morphology and cross-sectional characteristics of the coating were scrutinized using a Quanta 450FEG scanning electron microscope (SEM). Elemental distribution across the coating was assessed through energy-dispersive spectroscopy (EDS). A contact angle goniometer was employed to measure the wetting angles of the micro-arc oxidation coating samples, with five replicate measurements taken to determine the mean wetting angle. The potentio-dynamic polarization curves of the coating were recorded using a Keithley CS series all-in-one electrochemical workstation. The electrochemical setup involved a three-electrode configuration, with simulated body fluid (SBF) at 37 °C serving as the electrolyte for the polarization measurements (refer to Table 1). The working electrode comprised the micro-arc oxidation coating sample, with an exposed area of 1 cm^2^, a platinum plate acting as the counter electrode, and a saturated calomel electrode that served as the reference. Prior to initiating the test, the samples were immersed in SBF to allow for potential stabilization, conducted at a scanning rate of 1 mV/s.

## 3. Results and Discussion

### 3.1. SLM Sample Analysis

**Figure 2:**
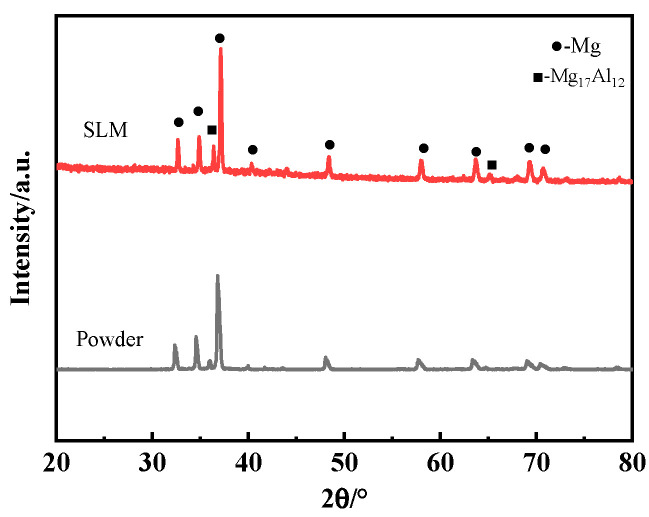
XRD patterns of AZ91 magnesium alloy powder and printed samples.

Figure 2 illustrates the XRD patterns of the AZ91 magnesium alloy in two forms: powder and printed samples. It is apparent that the powder and the SLM sample consist of α-Mg and β-Al12Mg17 intermetallic compounds. X-ray diffraction analysis revealed no new diffraction peaks in the samples processed via SLM. The intensity of the main peaks for the printed magnesium alloy samples is marginally higher than that of the powder form. The slightly enhanced main peak intensity in the printed samples, when compared to the powder, implies a greater intensity ratio of α-Mg to β-Al12Mg17 in the printed magnesium alloy [21].

**Figure 3:**
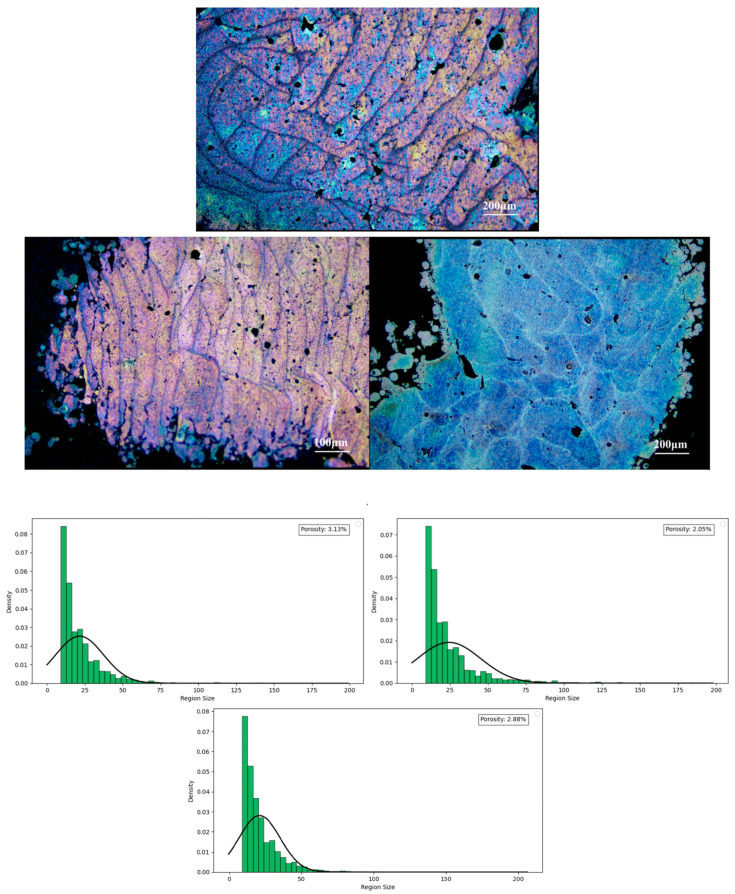
Microstructural images of 3D-printed magnesium alloy and analysis of surface porosity defects and density.

In the realm of additive manufacturing, a laser beam acts as the heat source, sequentially scanning the powder material in layers. The laser’s energy quickly melts the magnesium (Mg) powder, creating a molten pool. This pool rapidly cools and solidifies under the influence of the cooling effect from the support layer or other previously solidified layers [21]. The swift cooling phase induces the Mg within the molten pool to crystallize in a distinctive pattern, leading to the formation of a characteristic microstructure. The microstructure that emerges post-solidification of the molten pool displays a fish scale-like pattern, an outcome of the specific crystallization process during rapid cooling. The characteristics of this fish scale-like structure, including its morphology and dimensions, are indicative of the stability of the additive manufacturing process and the appropriateness of the selected process parameters. A closer examination of the fish scale-like structure, as depicted in Figure 3, reveals that the grain cross-sections within the molten pool take on elongated, strip-like, or slender columnar forms. The configuration and distribution of these grains are intricately linked to the solidification dynamics of the molten pool.

**Figure 4:**
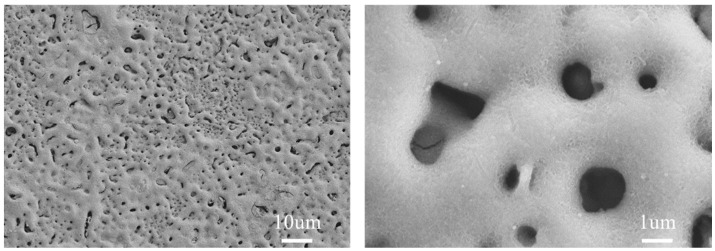
SEM surface morphology of SLM magnesium alloy micro-arc oxide film.

The microstructure of the MAO-treated Mg alloy sample is depicted in Figure 4, revealing a U-shaped melt pool morphology. The keyhole effect induces instability within the melt pool, culminating in the creation of keyholes at the pool’s base. It is widely recognized that magnesium (Mg) and zinc (Zn) elements have a high propensity for evaporation [22]. Should the alloy’s composition entrap vapor and the encircling inert argon gas fails to escape prior to the solidification of the melt pool, gas pores are formed.

### 3.2. Analysis of the Surface Micromorphology of the Film

Scanning electron microscopy (SEM) was utilized to examine the surface morphology of the micro-arc oxidation (MAO) film on the SLM magnesium alloy, as presented in Figure 4. The SEM images reveal that the film’s surface is populated with numerous micro-pores. The micro-pores’ distribution of the MAO film layer on the SLM magnesium alloy appears uneven, and the pore sizes are diverse. This uneven distribution and variation in size may stem from the presence of unfused pores on the SLM magnesium alloy’s surface. During the micro-arc oxidation process, these unfused pores become sites where arc discharge creates micro-pores with smaller diameters, a consequence of the smaller, unmelted powder particles within these pores that lead to the formation of narrower discharge channels. Additionally, the surface of the SLM magnesium alloy MAO film displays micro-cracks and areas of molten accumulation. These features arise as the MAO treatment duration extends, causing the arc discharge within the channels to produce high temperatures. Such temperatures melt the matrix and oxides, and the thermal energy propels the molten products to flow through the discharge channels, where they quickly solidify upon contact with the electrolyte, resulting in surface accumulations. Concurrently, the high temperatures from the arc discharge induce thermal stress within the film, which in turn leads to the formation of micro-cracks on the film’s surface.

**Figure 5:**
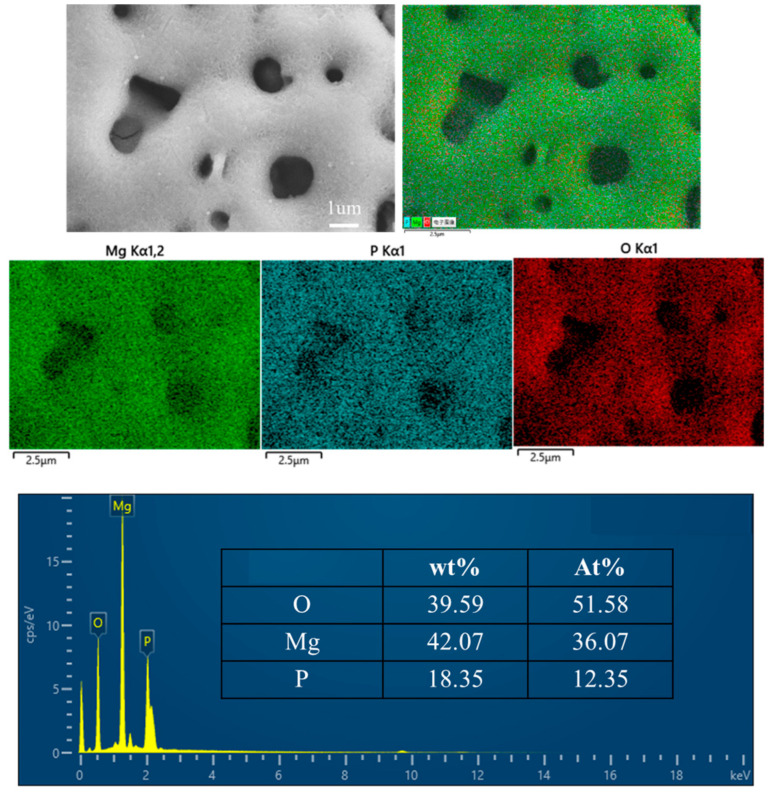
EDS spectra of SLM magnesium alloy micro-arc oxide coating.

Figure 5 presents the surface energy-dispersive spectroscopy (EDS) spectrum of the micro-arc oxidation (MAO) film on the SLM magnesium alloy. The elemental composition analysis by EDS reveals that the film is predominantly composed of oxygen (O), magnesium (Mg), and phosphorus (P). Initially, during the treatment, the film is largely composed of magnesium oxide (MgO), which is a result of the matrix’s inherent reaction. With an increase in treatment duration, the matrix engages in a reaction with phosphate ions (PO_4_^3−^) from the electrolyte, leading to the formation of magnesium phosphate (Mg_3_(PO_4_)_2_). The surface elemental composition of the MAO film, characterized by elevated levels of O and Mg, suggests a predominant presence of MgO. The relatively lower phosphorus content implies that a limited number of PO_4_^3−^ ions are integrated into the film-forming reaction throughout the growth process [23].

### 3.3. Surface Micromorphology Analysis of the Film Layer

**Figure 6:**
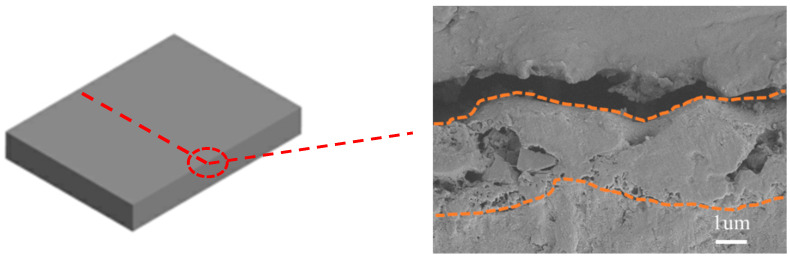
SEM cross-sectional view of the micro-arc oxide coating layer of SLM magnesium alloy. (The red dotted line shows the position of the cut material and the orange dotted line shows the membrane outline).

Figure 6 displays the SEM cross-sectional images of the micro-arc oxidation (MAO) film layers on the SLM magnesium alloy. The MAO film layer on the SLM magnesium alloy measures approximately 3.23 μm in thickness. Observations from the figure reveal that the MAO film layers on the SLM magnesium alloys possess micro-pores internally. At the interface between the MAO film and the substrate, the film layer on the SLM magnesium alloy appears relatively more porous. This suggests that the surface defects present on the SLM magnesium alloy influenced the early stages of the micro-arc oxidation process. Unmelted powder within these defects resulted in the formation of smaller discharge channels. Conversely, areas of the surface free from defects experienced typical micro-arc oxidation, leading to the accumulation of oxides and molten materials around these smaller channels. As the treatment duration extended, these channels became occluded by oxides and molten deposits, trapping the gases and consequently forming micro-pores within the film layer. This process also led to a more porous film layer at the interface with the substrate.

**Figure 7:**
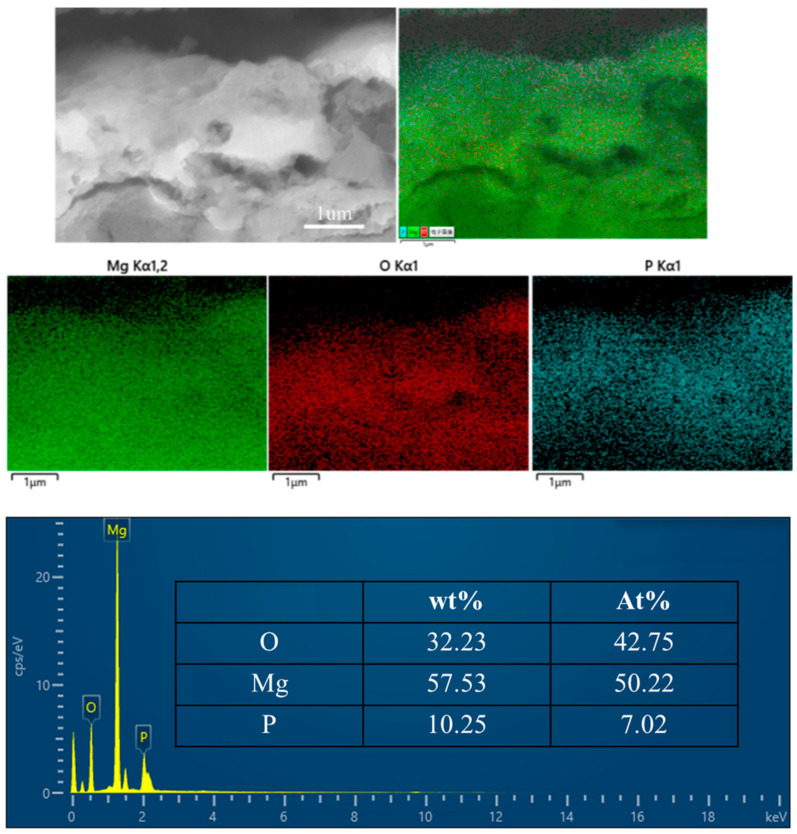
Cross-sectional EDS spectra of SLM magnesium alloy micro-arc oxide coating.

Figure 7 presents the cross-sectional energy-dispersive spectroscopy (EDS) spectrum and the elemental distribution within the micro-arc oxidation (MAO) film on the SLM magnesium alloy. The elemental distribution derived from the spectrum confirms that the film is predominantly composed of oxygen (O), magnesium (Mg), and phosphorus (P). Phosphorus constitutes 10.25% of the film’s cross-sectional area, in contrast to 18.35% on the surface. This disparity in phosphorus content between the cross-section and the surface implies a reduced abundance of the Mg_3_(PO_4_)_2_ phase within the film’s interior. The increased presence of oxygen and magnesium in the cross-section suggests that the film’s core is primarily the MgO phase. This phenomenon can be attributed to the initial stage of the reaction, where a MgO film layer forms on the magnesium alloy matrix as a result of passivation. Upon reaching the breakdown voltage as the applied voltage increases, the oxide film’s weaker points electrically break down, producing heat and causing the formation of molten products. These products, predominantly Mg_3_(PO_4_)_2_, are propelled to the film’s surface through the discharge channels. Consequently, the Mg_3_(PO_4_)_2_ phase is less prevalent within the film and is mainly concentrated on the surface. As the treatment time extends, the film’s thickness increases. Once the thickness surpasses a critical threshold, the voltage can no longer breach the oxide film, halting the micro-arc oxidation process and preserving the internal MgO phase within the film [24].

### 3.4. Phase Composition of the Film

**Figure 8:**
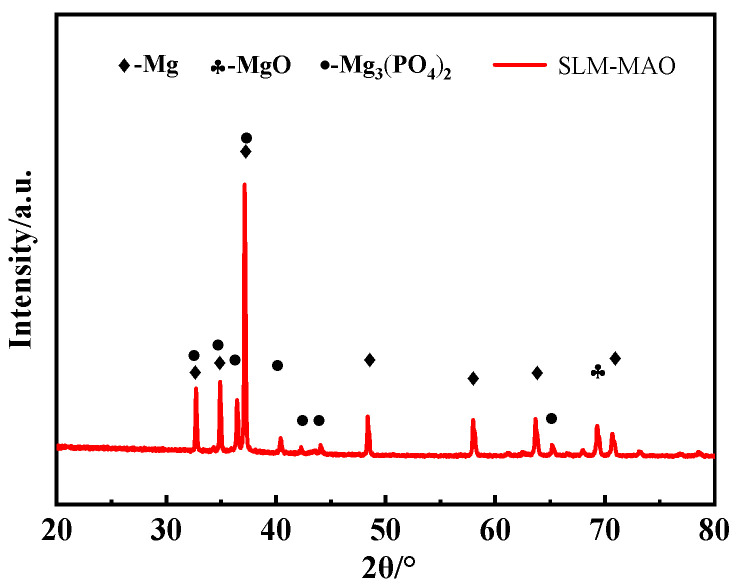
XRD pattern of micro-arc oxide coating layer on SLM magnesium alloy.

Figure 8 illustrates the X-ray diffraction (XRD) spectrum of the micro-arc oxidation (MAO) film applied to the SLM magnesium alloy. The film’s composition is predominantly magnesium oxide (MgO) and magnesium phosphate (Mg_3_(PO_4_)_2_). Given the film’s thin profile, high-intensity peaks corresponding to the Mg phase are noticeable within the oxide layer. The emergence of these distinct phases can be elucidated by the following reactions [25]:

(1) $${Mg}^{2+}+2{OH}^{-}\to Mg{\left(OH\right)}_{2}\to MgO+H_{2}O\;$$

(2) $$3{Mg}^{2+}+2{PO}_{4}^{3-}\to {Mg}_{3}{\left({PO}_{4}\right)}_{2}$$

In conjunction with the EDS analysis of the film’s surface, the elemental distribution within the SLM magnesium alloy MAO film indicates that the film’s surface is predominantly the MgO phase. This observation is attributed to the presence of defects associated with the printing process on the surface and within the SLM magnesium alloy, which can disrupt the breakdown of discharge channels and, in turn, affect the formation of molten products. As a result, the formation of magnesium phosphate (Mg_3_(PO_4_)_2_) is impacted, leading to a reduced abundance of phosphorus (P). This indicates that the micro-arc oxidation process on the SLM magnesium alloy is significantly influenced by the material’s inherent characteristics, including defects introduced during the printing process. These defects have the potential to modify the film’s uniformity and the distribution of its phases.

### 3.5. Analysis of Wetting Angle of Micro-Arc Oxide Film Layer

**Figure 9:**
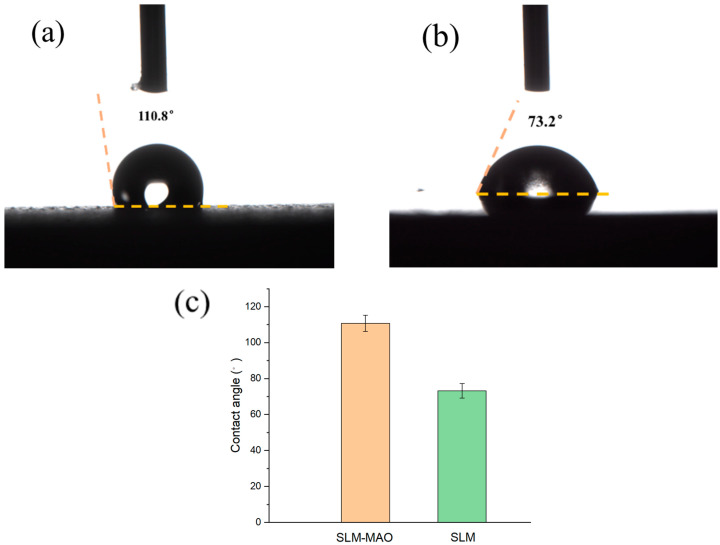
Wetting angle (**a**) of SLM magnesium alloy before and after micro-arc oxidation treatment; (**b**) post-processing; (**c**) Comparison of wetting angle.

The surface of an implant can be characterized as either hydrophobic or hydrophilic, a property that significantly influences the interaction with human tissue. Hydrophilicity plays a pivotal role in cell proliferation and adhesion, making it a critical attribute for implant materials. The hydrophilic or hydrophobic nature of the micro-arc oxidation (MAO) coating is ascertained by measuring the contact angle formed when water interacts with the coating’s surface. A material is deemed hydrophilic if the contact angle is below 90°, and hydrophobic if it exceeds 90° [26]. Figure 9 presents the contact angle images of the SLM magnesium alloy both prior to and following MAO treatment. The contact angle was measured at 110.8° before treatment and reduced to 73.2° post-treatment. These findings indicate that the 3D-printed magnesium alloy’s contact angle decreases after MAO treatment, inferring an enhanced hydrophilicity. This outcome is attributed to the formation of a porous film on the alloy’s surface due to MAO, which, due to its porosity, results in a lower contact angle and consequently improved hydrophilic properties.

### 3.6. Analysis of Corrosion Resistance of Micro-Arc Oxide Coating

**Figure 10:**
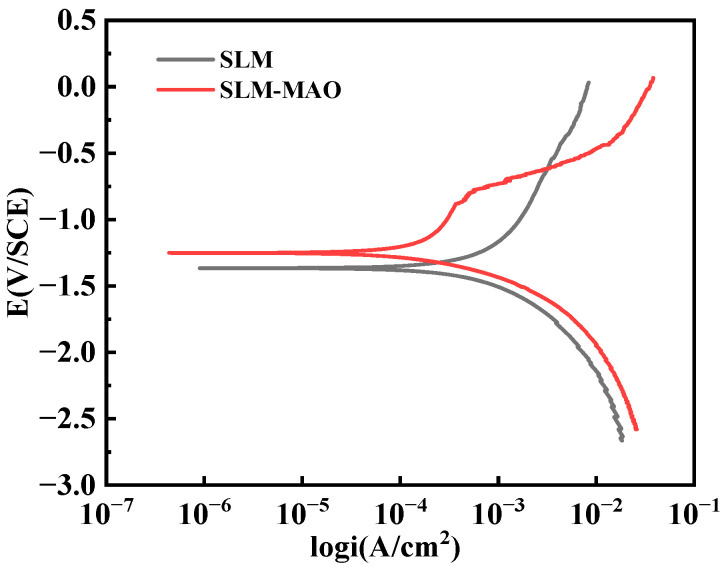
Kinetic polarization curves measured in SBF simulated body fluids before and after microarc oxidation of SLM magnesium alloys.

The corrosion resistance of micro-arc oxidation coatings, prepared using three distinct electrolytes, was evaluated utilizing potentiodynamic polarization curves. Figure 10 illustrates these polarization curves for the SLM magnesium alloy substrate both before and after undergoing micro-arc oxidation treatment in simulated body fluid (SBF). The derived fitting results of the polarization curves are detailed in Table 2, which includes the corrosion potential (E_corr) and the corrosion current density (I_corr). The polarization resistance (R_p) is calculated employing the following Stern–Geary equation [26]:

(3) $$R_{p}=\frac{b_{a}\times b_{c}}{2.303I_{corr}\left(b_{a}+b_{c}\right)}$$

Upon examining the parameter values listed in Table 2, it is evident that the corrosion potential (E_corr) for the SLM magnesium alloy that has undergone micro-arc oxidation treatment exceeds that of the untreated alloy. Concurrently, the corrosion current density (I_corr) post-treatment is observed to be lower than the pre-treatment values. These observations indicate that the micro-arc oxidation treatment effectively enhances the corrosion resistance of the SLM magnesium alloy substrate. The corrosion resistance of the SLM magnesium alloy is thus deemed to be superior following micro-arc oxidation treatment compared to the untreated state. Despite an improvement in corrosion resistance, the polarization resistance (R_p) values still fall within a similar order of magnitude. This is likely due to the presence of inherent defects within the SLM magnesium alloy samples, which result in a non-uniform distribution of pore sizes across the film layer and the formation of micro-cracks. Furthermore, defects such as unmelted powder particles from the SLM process contribute to a more porous film layer during the micro-arc oxidation treatment. These factors collectively impose a limit on the extent of corrosion resistance enhancement that the micro-arc oxidation film layer can provide to the substrate.

### 3.7. AFM Analysis of Micro-Arc Oxide Coatings

**Figure 11:**
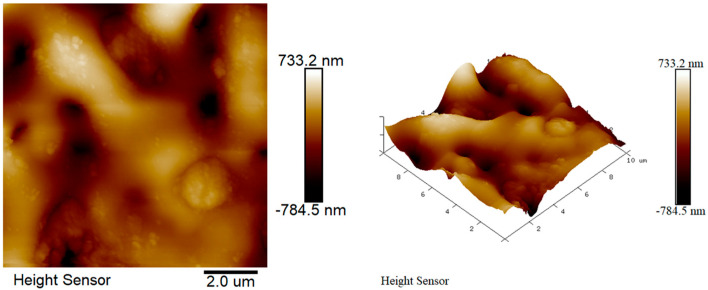
AFM image of 3D-printed magnesium alloy micro-arc oxide coating.

Figure 11 displays the atomic force microscopy (AFM) images of the micro-arc oxidation film for the 3D-printed magnesium alloy. The roughness of the MAO film layer of the 3D-printed magnesium alloy is 180 nm. The two-dimensional AFM images highlight the presence of distinct pores on the surface, characterized by numerous protrusions and depressions, which signify considerable variations in surface topography. The computed average surface roughness, determined through software analysis, is 180 nm, exceeding that of the phosphate system film layer applied to the magnesium alloy as previously discussed. The increased roughness observed in the 3D-printed magnesium alloy is predominantly attributed to surface defects that result in non-uniform discharge patterns during the micro-arc oxidation treatment. Corroborated by SEM imaging, the presence of micro-cracks on the surface further contributes to the heightened roughness of the micro-arc oxidation film layer on the 3D-printed magnesium alloy. An elevated level of roughness can, to a certain degree, augment cell adhesion and proliferation. The surface characteristics of the micro-arc oxidation film, including its roughness and porosity, play a pivotal role in the interaction with biological tissues. Enhanced surface roughness can potentially offer an increased surface area and a greater number of cellular binding sites, which may facilitate cell attachment and growth. Nonetheless, it is imperative to ensure that these surface features remain biocompatible and do not elicit any adverse reactions within the biological environment.

**Figure 12:**
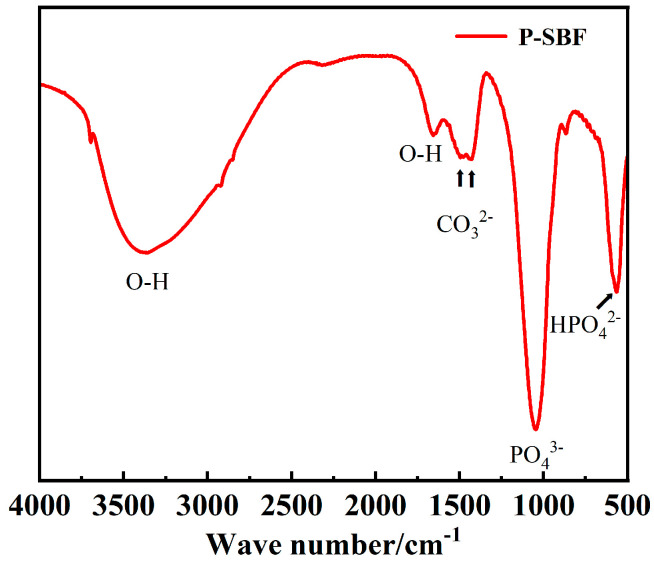
The infrared spectroscopy analysis of the MAO coating after immersion in SBF for 21 days.

Figure 12 shows the infrared spectroscopy analysis of the MAO coating after immersion in SBF for 21 days. The groups mainly include PO_4_^3−^, CO_3_^2−^, OH^−^, etc. which correspond to calcium–phosphate compounds, analogous to hydroxyapatite. The 637 cm^−1^ peak corresponds to the CO_3_^2−^ root. The 1020–1070 cm^−1^ region corresponds to the stretching vibration of the PO_4_^3−^ root. The 3550–3700 cm^−1^ region corresponds to the OH^−^ group [27]. The result indicates that the coating surface has experienced chemical transformations, culminating in the formation of these compounds. Hydroxyapatite (HA), a key component of bone minerals, is renowned for its exceptional biocompatibility and its ability to osseointegrate. Throughout the immersion in SBF, the MAO coating engages in interactions with the fluid’s ions, undergoing ion exchange and chemical reactions that culminate in the deposition of calcium–phosphate compounds akin to hydroxyapatite.

In addition to phosphate ions (PO_4_^3−^), the detection of carbonate (CO_3_^2−^) and hydrogen phosphate (HPO_4_^2−^) functional groups provides further evidence of the chemical transformations that the film layer undergoes during its immersion. The carbonate ions (CO_3_^2−^) likely stem from the carbonates naturally found in the simulated body fluid (SBF) or result from the film layer’s reaction with atmospheric carbon dioxide. Conversely, hydrogen phosphate ions (HPO_4_^2−^) may arise as byproducts of phosphate hydrolysis or ionization processes under certain conditions. As the phosphate-based film layer immerses in SBF, its surface participates in intricate chemical reactions that involve calcium ions, phosphate ions, and a spectrum of other ions within the fluid. These interactions culminate in the precipitation of calcium–phosphate compounds resembling hydroxyapatite, which subsequently modifies the film’s chemical makeup and may subsequently augment its performance.

## 4. Summary

This study explores the impact of surface morphology, elemental distribution, and phase composition of the micro-arc oxidation (MAO) film on the hydrophilicity and corrosion resistance of magnesium alloys fabricated using Selective Laser Melting (SLM) technology. The findings lead to the following conclusions:

(1) The SLM magnesium alloy exhibits surface porosity defects ranging from 2% to 3.2%, which significantly influence the morphology and functionality of the resulting film layer formed during the micro-arc oxidation process. These defects manifest as pores on the surface, leading to an uneven distribution of micropores with varying sizes across the layer. The surface roughness of the micro-arc oxidation (MAO) film layer on the 3D-printed magnesium alloy exhibits a higher roughness of 180 nanometers. The presence of unfused pores and unmelted powder particles on the SLM magnesium alloy’s surface results in the formation of small discharge channels on the powder particles, consequently producing micro-pores with reduced diameters. This phenomenon results in an uneven distribution of micro-pores and a variation in pore sizes within the MAO film layer of the SLM magnesium alloy.
(2) The film’s composition is predominantly magnesium oxide (MgO) and magnesium phosphate (Mg_3_(PO_4_)_2_). The film’s higher concentration of magnesium (Mg) and oxygen (O) elements, in contrast with the lower phosphorus (P) content, indicates that MgO is the predominant phase. The increased P content on the film’s surface relative to its interior implies that Mg_3_(PO_4_)_2_ formation predominantly occurs on the film’s surface.
(3) The application of the micro-arc oxidation technique enhances the hydrophilicity of SLM-fabricated magnesium alloys, thereby potentially conferring bioactivity. Post-MAO treatment, both the corrosion resistance and surface roughness of the SLM magnesium alloy are significantly improved. Nonetheless, the existence of certain defects within the SLM magnesium alloy samples adversely affects the enhancement of corrosion resistance.

The phosphate system electrolyte proves to be highly effective in promoting the deposition of calcium and phosphorus within the micro-arc oxidation coating, specifically in the simulated body fluid (SBF) environment. When this electrolyte is utilized for the micro-arc oxidation of SLM magnesium alloy, it significantly enhances both the biocompatibility and corrosion resistance of the alloy. Consequently, these findings hold immense potential as a valuable reference for the surface modification of 3D-printed magnesium alloy implants, aiming to achieve optimal biocompatibility and superior corrosion resistance.

## Figures and Tables

**Table 1:** Formula for preparing 1 liter of simulated body fluid (SBF).

Numbering	Reagent	Content	Purity
1	NaCl	8.035 g	99.5%
2	NaHCO_3_	0.355 g	99.5%
3	KCl	0.225 g	99.5%
4	K_2_HPO_4_·3H_2_O	0.231 g	99.0%
5	MgCl_2_·6H_2_O	0.311 g	98.0%
6	HCl	39.0 mL	—
7	CaCl_2_	0.292 g	95.0%
8	Na_2_SO_4_	0.072 g	99.0%
9	Tris	6.118 g	99.0%
10	HCl	0~5 mL	—

**Table 2:** Fitting results of potential polarization curves measured in SBF simulated body fluids before and after microarc oxidation treatment of SLM magnesium alloys.

Sample	$E_{corr}$/V	$I_{corr}$/(A·cm^2^)	$R_{p}$/(Ω·cm^2^)
SLM	−1.37	4.08 × 10^−4^	3.80 × 10^2^
SLM-MAO	−1.25	1.61 × 10^−4^	6.09 × 10^2^

## Data Availability

Data are contained within the article.
